# Suppressed autophagic response underlies augmentation of renal ischemia/reperfusion injury by type 2 diabetes

**DOI:** 10.1038/s41598-017-05667-5

**Published:** 2017-07-13

**Authors:** Shingo Muratsubaki, Atsushi Kuno, Masaya Tanno, Takayuki Miki, Toshiyuki Yano, Hirohito Sugawara, Satoru Shibata, Koki Abe, Satoko Ishikawa, Kouhei Ohno, Yukishige Kimura, Yuki Tatekoshi, Kei Nakata, Wataru Ohwada, Masashi Mizuno, Tetsuji Miura

**Affiliations:** 1 Department of Cardiovascular, Renal and Metabolic Medicine, Sapporo Medical University School of Medicine, Sapporo, Japan; 2 Department of Pharmacology, Sapporo Medical University School of Medicine, Sapporo, Japan

## Abstract

Diabetes mellitus is a major risk factor for acute kidney injury (AKI). Here, we hypothesized that suppression of autophagic response underlies aggravation of renal ischemia/reperfusion (I/R) injury by type 2 diabetes mellitus (T2DM). In OLETF, a rat model of T2DM, and its non-diabetic control, LETO, AKI was induced by unilateral nephrectomy and 30-min occlusion and 24-h reperfusion of the renal artery in the contralateral kidney. Levels of serum creatinine and blood urea nitrogen and tubular injury score after I/R were significantly higher in OLETF than in LETO. Administration of chloroquine, a widely used autophagy inhibitor, aggravated I/R-induced renal injury in LETO, but not in OLETF. In contrast to LETO, OLETF exhibited no increase in autophagosomes in the proximal tubules after I/R. Immunoblotting showed that I/R activated the AMPK/ULK1 pathway in LETO but not in OLETF, and mTORC1 activation after I/R was enhanced in OLETF. Treatment of OLETF with rapamycin, an mTORC1 inhibitor, partially restored autophagic activation in response to I/R and significantly attenuated I/R-induced renal injury. Collectively, these findings indicate that suppressed autophagic activation in proximal tubules by impaired AMPK/ULK1 signaling and upregulated mTORC1 activation underlies T2DM-induced worsening of renal I/R injury.

## Introduction

Diabetes mellitus is a major risk factor for kidney damage. Diabetic nephropathy is a microvascular complication and is the most common cause of end-stage renal disease in many countries. In addition, patients with diabetes mellitus have increased risk for acute kidney injury (AKI), a condition of abrupt decline in kidney function by renal or extra-renal causes^1, 2^. Since the development of AKI is associated with poor prognosis in critically and non-critically sick patients^3, 4^, prevention of AKI in patients with AKI risk factors, including diabetes mellitus, is an important clinical issue. Studies using animal models of type 1 diabetes (T1DM) and type 2 diabetes (T2DM) have demonstrated that renal damage after ischemia/reperfusion (I/R), a main cause of AKI^5^, was enhanced by diabetes^6–8^. However, the mechanism by which diabetes increases susceptibility to AKI has not been fully elucidated.

Autophagy is a cellular process in which cytoplasmic components and damaged organelles are degraded in autolysosomes to recycle them as energy sources and to maintain protein quality and cellular homeostasis^9–11^. It has been reported that podocyte-specific deletion of Atg5, an essential autophagy gene, enhanced age-dependent glomerulosclerosis^12^. Deficiency of Atg5 in proximal tubules was shown to induce tubular degeneration^13^. These findings indicate crucial roles of autophagy in maintaining homeostasis of both the podocyte and the tubular cell. In addition to its house-keeping function, autophagy responds to various cellular stresses^11^. Podocyte-specific deletion of Atg5 promoted diabetes-induced glomerulopathy in murine models of T1DM^14^ and T2DM^15^, indicating that autophagy in the podocyte counteracts metabolic stress by diabetes. I/R activates autophagy in tubular cells^13, 16–19^, and inhibition of the autophagy by an inhibitor such as chloroquine (CQ) or 3-methyladenine^17^ or by deletion of Atg5^13, 18^ or Atg7^20^ in proximal tubular cell aggravated renal damage after I/R in mice. Thus, autophagy activated by I/R appears to have a protective role against I/R-induced renal damage.

A few lines of evidence indicate that diabetes mellitus impairs autophagy^21^. We recently found that myocardial autophagy in response to heart failure by acute myocardial infarction (AMI) was attenuated in a rat model of T2DM and that the impaired autophagic response was associated with increased mortality after AMI^22^. Basal autophagy in renal tubular cells was reported to be suppressed in Wistar fatty rats^23^. Autophagic activity in podocytes was found to be increased in mice with streptozotocin-induced diabetes before the development of glomerular lesions, but the activity decreased when mice exhibited glomerular lesions^14, 24, 25^. Diet-induced obesity reportedly suppressed proteinuria-induced activation of autophagy in the proximal tubular cells of mice^26^. However, it remains unclear whether activation of autophagy in tubular cells in response to I/R is impaired by diabetes and whether such an impairment, if any, contributes to diabetes-induced increase in renal susceptibility to I/R injury. To address these issues, we examined differences in renal autophagy and I/R-induced AKI between Otsuka Long-Evans Tokushima Fatty rats (OLETF), a model of T2DM, and non-diabetic controls, Long-Evans Tokushima Otsuka rats (LETO). In addition, the relationship between autophagy and AKI in OLETF was examined by pharmacological manipulation of autophagic flux.

## Results

### Basal renal status in LETO and OLETF

OLETF at the age of 25–30 weeks were heavier than LETO and their levels of fasting blood glucose and glycoalbumin were significantly higher than those in age-matched control LETO (Table 1). Serum creatinine and blood urea nitrogen (BUN) levels were not increased in OLETF at this age compared with the levels in LETO (Table 1). However, consistent with previous reports^27, 28^, OLETF showed higher urine albumin/creatinine and urine protein/creatinine ratios and greater kidney weight than those in LETO (Table 1). In addition, periodic acid-Schiff (PAS) staining showed that the mesangial areas tended to be increased and that the glomerular areas were significantly enlarged in OLETF compared with those in LETO (Supplementary Fig. 1). These findings indicate that OLETF had developed overt nephropathy at this age.

**Table 1:** Baseline parameters in LETO and OLETF.

	LETO (N = 6)	OLETF (N = 6)
Body weight (g)	505 ± 11	609 ± 12*
Blood glucose (mg/dl)	114 ± 5	240 ± 33*
Glycoalbumine (%)	10.6 ± 0.3	14.2 ± 3.5*
Serum creatinine (mg/dl)	0.61 ± 0.04	0.41 ± 0.02*
Blood urea nitrogen (mg/dl)	15.8 ± 1.0	13.9 ± 0.5
Urine albumin/creatinine ratio (g/g)	0.61 ± 0.23	1.79 ± 0.40*
Urine protein/creatinine ratio (g/g)	0.62 ± 0.20	6.36 ± 2.43*
Kidney weight (g)	1.23 ± 0.04	1.88 ± 0.07*

Values are means ± SEM.

*p < 0.05 vs. LETO.

### Renal damage after ischemia/reperfusion in LETO and OLETF with and without autophagy inhibitor

We assessed renal damage after I/R in LETO and OLETF and also the effect of autophagy inhibition on I/R-induced renal damage by using chloroquine (CQ, 10 mg/kg/day), a widely used inhibitor of autophagy^17, 22, 29–31^. LETO (n = 13), OLETF (n = 12), CQ-treated LETO (LETO+CQ, n = 9), and CQ-treated OLETF (OLETF+CQ, n = 10) underwent nephrectomy of the right kidney followed by 30-min ischemia/24 h-reperfusion in the left kidney. We employed this one-kidney one-I/R model as in earlier studies^32–34^ because, in pilot experiments, bilateral renal I/R resulted in a large data variation between experiments in our hands (data not shown). The unilateral renal I/R method has an advantage that the right kidney removed before I/R of the contralateral kidney serves as control tissue before I/R. Mortality at 24 h after surgery was 0% in LETO, OLETF and CQ-treated OLETF, and one rat in the LETO+CQ group died of an unknown cause just after the surgery. One LETO was excluded from analyses because reperfusion was failed due to a technical problem.

**Figure 1:**
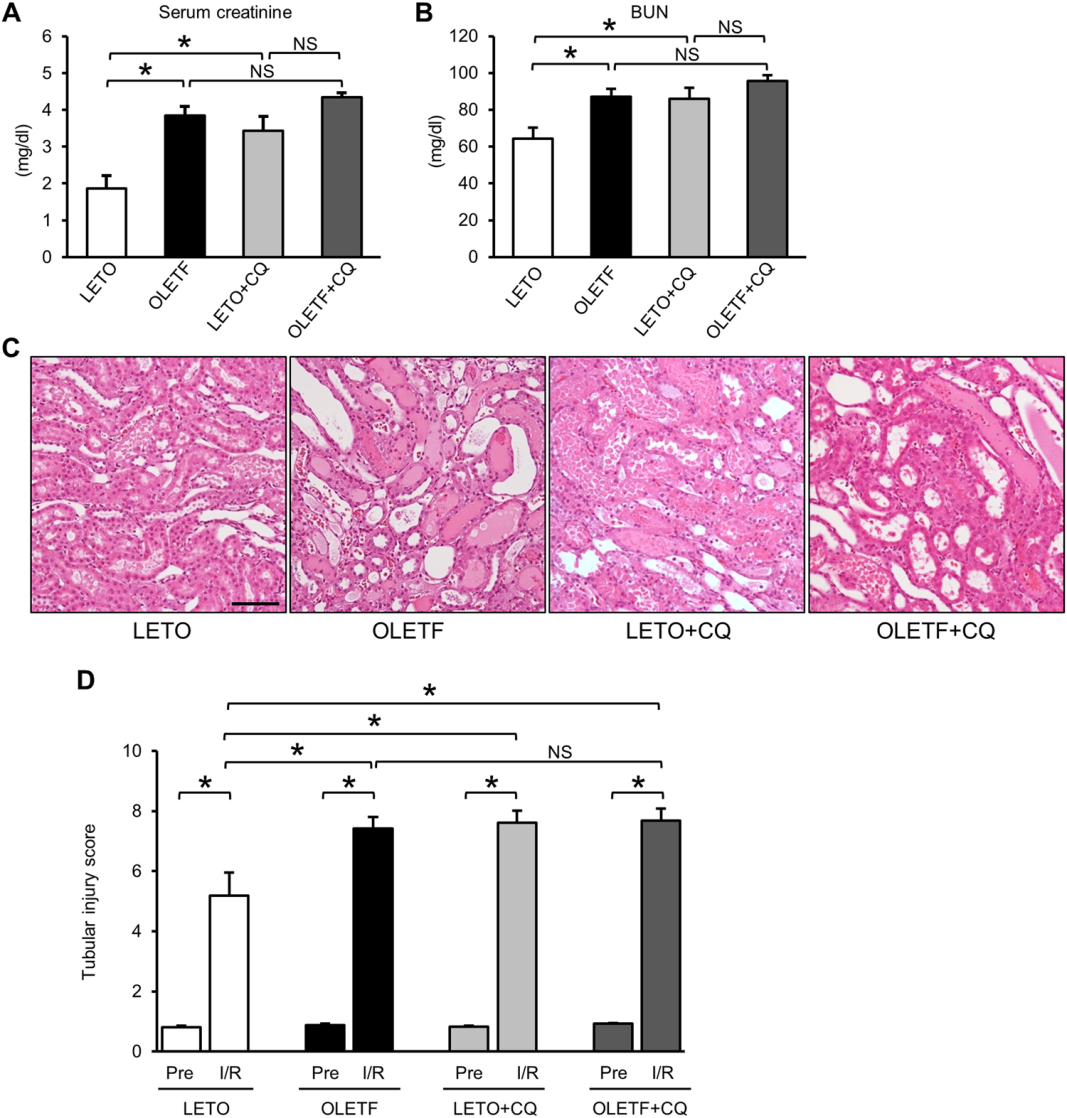
Type 2 diabetes and chloroquine increased susceptibility of the kidney to ischemia/reperfusion injury. Serum creatinine (**A**) and blood urea nitrogen (BUN) (**B**) levels of LETO (N = 12), OLETF (N = 12), LETO treated with chloroquine (LETO+CQ, N = 8), and OLETF treated with chloroquine (OLETF+CQ, N = 10) at 24 h after ischemia and reperfusion. (**C**) Representative images of hematoxylin-eosin staining of renal tissues at 24 h after ischemia/reperfusion. (**D**) Tubular injury score of renal tissues of before ischemia (Pre) and 24 h after ischemia/reperfusion (I/R) in the four groups. *P < 0.05. Scale bar, 100 μm. NS = not significant.﻿

Serum creatinine and BUN levels were 1.86 ± 0.35 mg/dl and 64.3 ± 6.0 mg/dl, respectively, in LETO after I/R (Fig. 1A and B). In OLETF, levels of serum creatinine (3.84 ± 0.25 mg/dl) and BUN (87.2 ± 4.1 mg/dl) after I/R were significantly higher than those in LETO after I/R. CQ treatment in LETO also increased levels of both serum creatinine (3.43 ± 0.39 mg/dl) and BUN (83.0 ± 5.9 mg/dl) after I/R compared with those of LETO (Fig. 1A and B). In contrast, pretreatment with CQ did not further increase levels of serum creatinine and BUN (4.34 ± 0.12 mg/dl, and 95.5 ± 3.4 mg/dl, respectively) in OLETF (Fig. 1A and B). Although tubular injury scores before I/R were comparable among the groups, the score after I/R was significantly higher in OLETF and LETO+CQ than in LETO (Fig. 1C and D). CQ did not significantly change tubular injury score in OLETF (Fig. 1C and D). Heart rate at 24 h after I/R under anesthesia was lower in OLETF than in LETO, and CQ treatment decreased heart rate at 24 h after I/R in LETO, but not in OLETF (Supplementary Table 1). These findings indicate that diabetes aggravates I/R-induced renal injury and confirmed that autophagic activation in response to renal I/R is renoprotective as previously reported^13, 17, 18, 20^. In addition, absence of significant aggravation of renal injury by CQ in OLETF suggests that renoprotective autophagy is substantially suppressed in OLETF.

### Impaired activation of autophagy in tubular cells in response to I/R in OLETF

**Figure 2:**
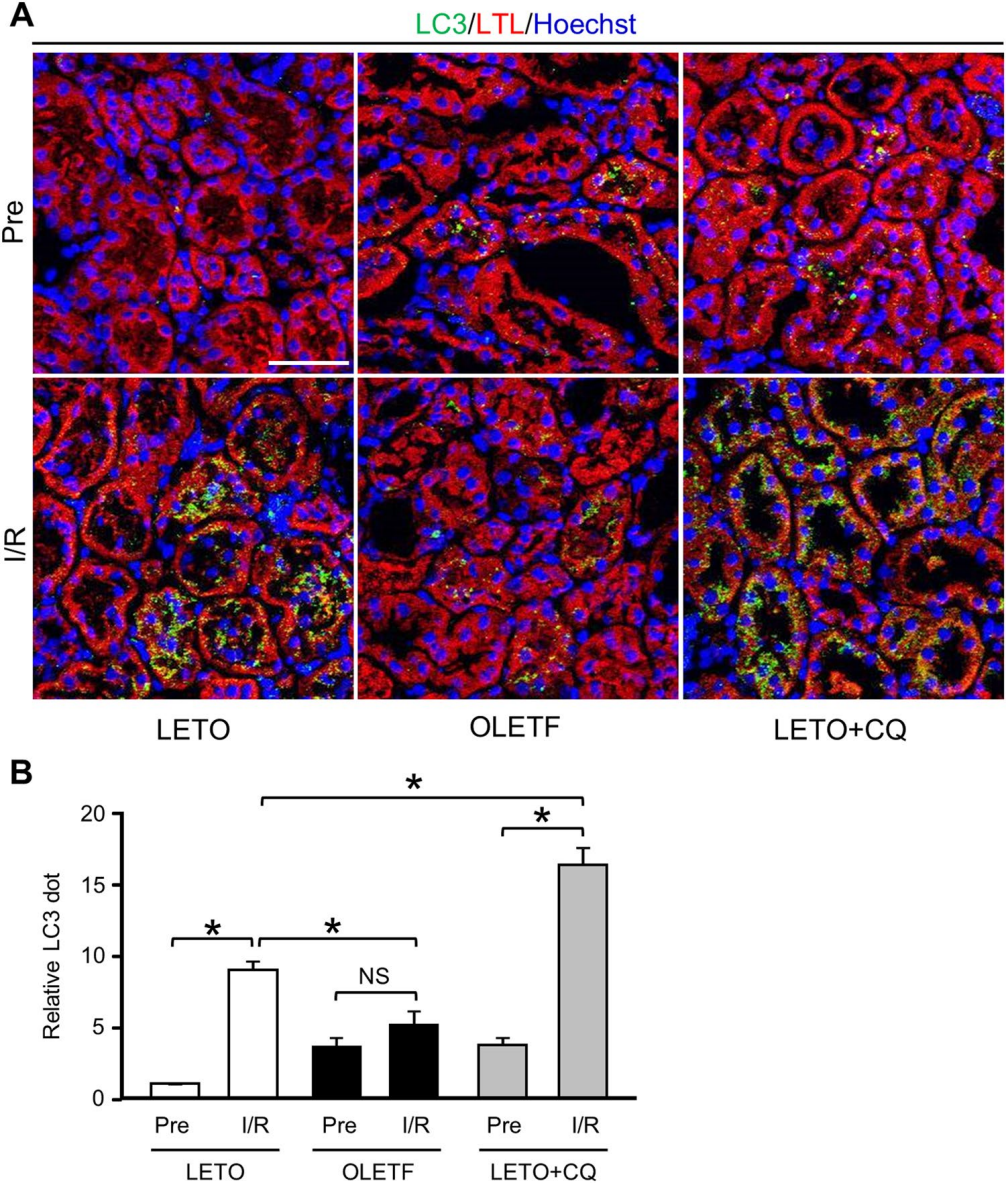
Autophagic response in proximal tubular cells after ischemia/reperfusion was attenuated in OLETF. (**A**) Representative immunofluorescence images of LC3 (green) and the proximal tubular marker lotus tetragonolobus lectin conjugated with Texas Red (LTL; red) in the kidneys of LETO, OLETF, and LETO treated with chloroquine (LETO+CQ). Nuclei were stained with Hoechst33342. Pre: pre-ischemia, I/R: 24 h after ischemia/reperfusion. (**B**) Summary data of LC3 dot numbers. N = 4 in each group. *P < 0.05, NS = not significant. Scale bar, 50 μm.

We next examined whether autophagic activation in response to I/R is altered in OLETF. In immunofluorescence analysis for LC3, LC3 dots were detected in proximal tubular cells that were labeled by *Lotus tetragonolobus* lectin (LTL) conjugated with Texas Red (Fig. 2A). Before I/R, the number of LC3 dots tended to be larger in OLETF than in LETO. CQ pretreatment increased LC3 dots at baseline in LETO, indicating accumulation of autophagosomes by inhibited lysosome function. I/R significantly increased LC3 dots in LETO but not in OLETF (Fig. 2B). The I/R-induced increase in LC3 dots was enhanced in the LETO+CQ group (Fig. 2B).

**Figure 3:**
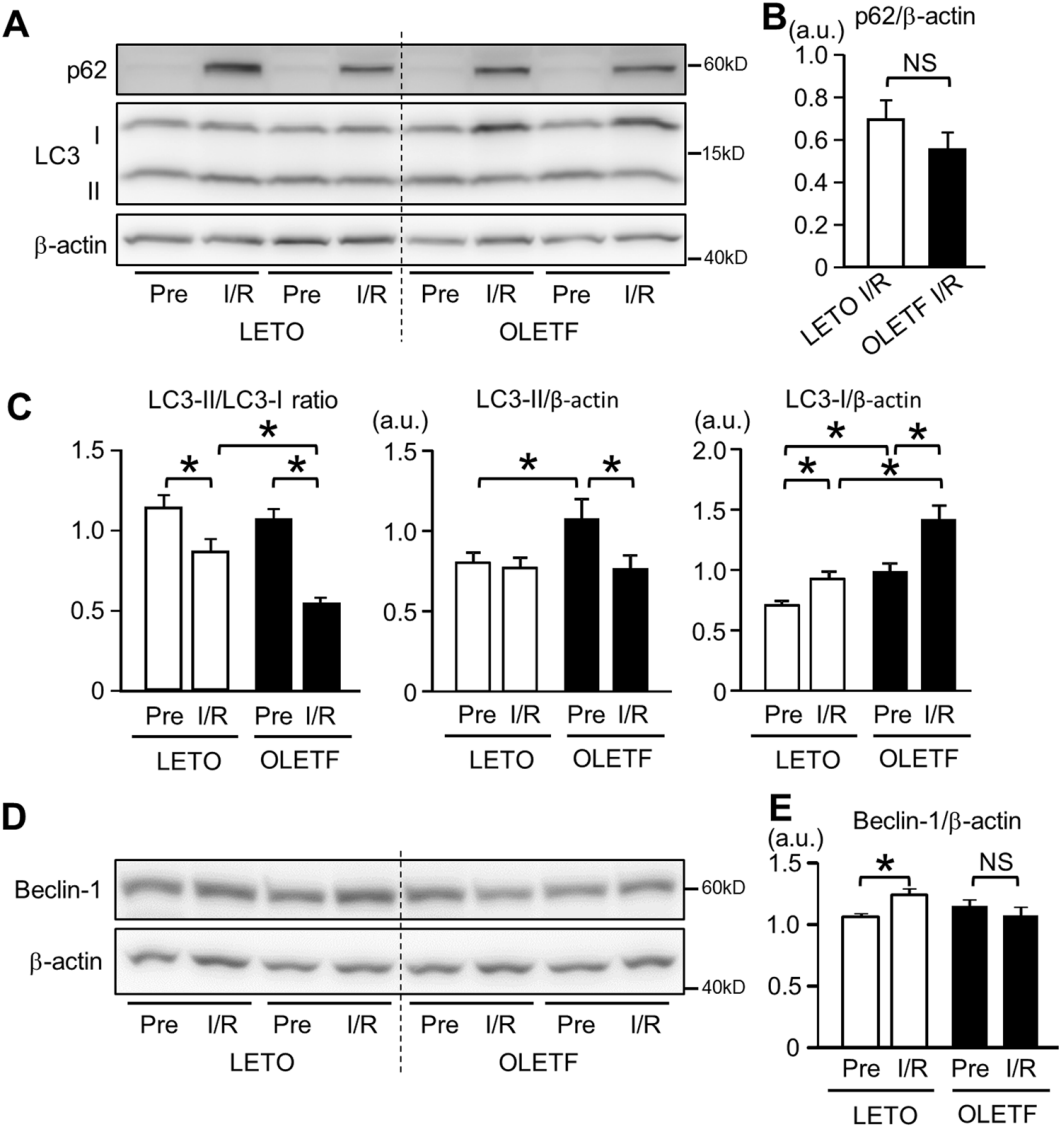
Activation of renal autophagy after ischemia/reperfusion was reduced in OLETF. (**A**) Representative immunoblot images for p62 and LC3 in the kidney. Summary data for p62 (**B**) and LC3-II/LC3-I ratio, LC3-II, and LC3-I (**C**) in total kidney tissues before ischemia (Pre) and 24 h after ischemia/reperfusion (I/R). Ratio of LC3-II to LC3-I after I/R was significantly reduced in OLETF. Representative immunoblots for beclin-1 (**D**) and summary data (**E**) in kidney tissues. Beclin-1 protein level was increased after I/R only in LETO. N = 8 in each group. *P < 0.05, NS = not significant.

Immunoblot analysis showed that LC3-II/LC3-I ratio, an index of LC3-I to LC3-II conversion, was rather decreased after I/R due to an increase in LC3-I level (Fig. 3A and C) in both LETO and OLETF. The level of LC3-II, an autophagosome marker, was not changed by I/R in LETO, and the LC3-II levels after I/R were similar in LETO and OLETF (Fig. 3A and C). These immunoblot results are apparently inconsistent with the results of immunofluorescence analyses (Fig. 2). The inconsistency may be due to the fact that whole kidney tissue lysates were used for immunoblotting. Nevertheless, LC3-II/LC3-I ratio after I/R was lower in OLETF than in LETO (Fig. 3A and C), suggesting attenuated autophagic response in OLETF. Protein levels of p62, a marker of autophagic activity^35^, were similarly increased after I/R in both LETO and OLETF (Fig. 3A and B). The protein level of beclin-1, a key regulator of autophagy as well as an autophagy marker in the kidney^36, 37^, was significantly upregulated after I/R in LETO but not in OLETF (Fig. 3D and E).

### Alterations in signals that regulate autophagy in OLETF

**Figure 4:**
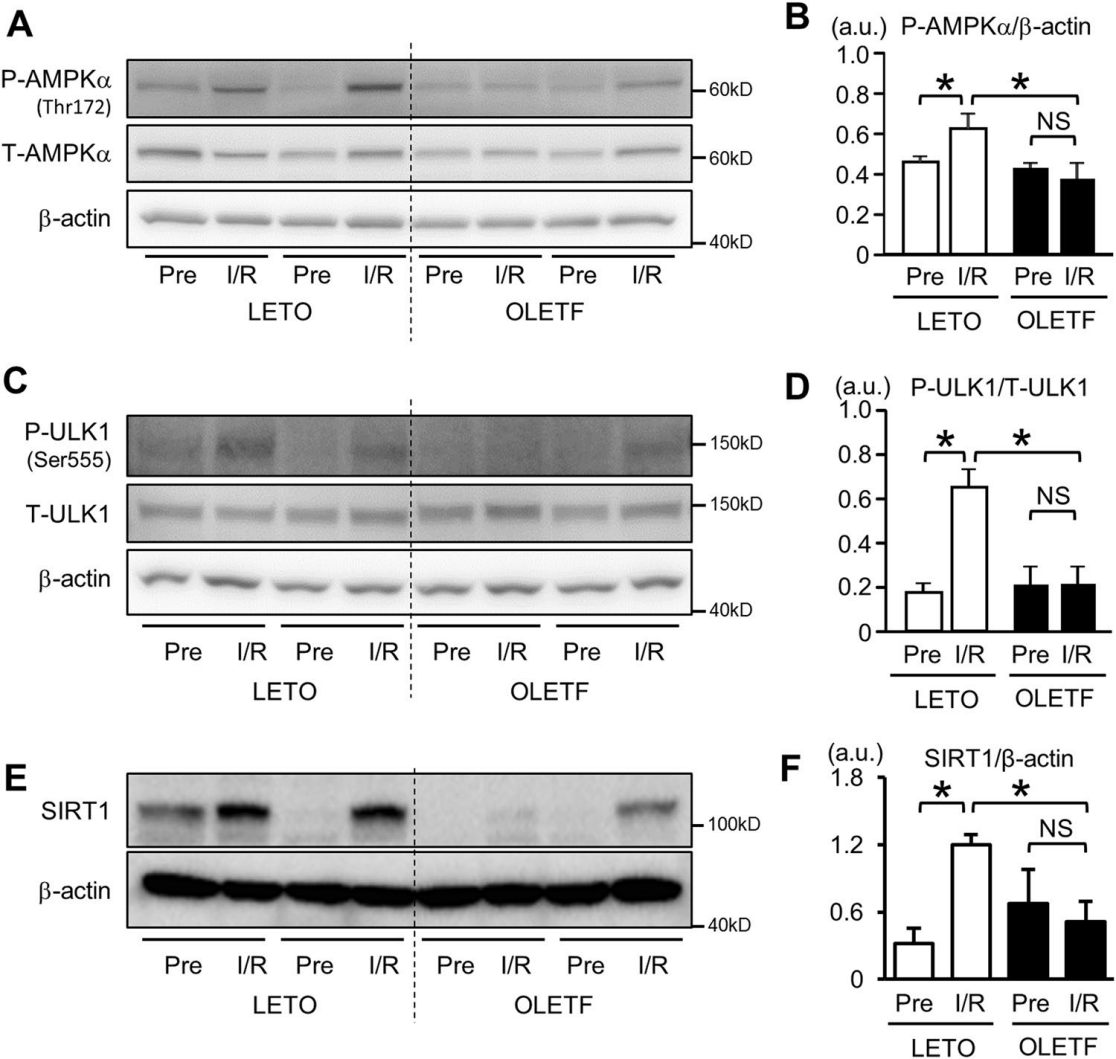
Analysis of AMPK/ULK1 signal pathways. (**A**) Representative immunoblots for phospho-Thr172 and total AMPKα in renal tissues before ischemia and 24 h after reperfusion in LETO and OLETF. Pre: pre-ischemia, I/R: 24 h after ischemia/reperfusion. (**B**) Summary data for phospho-Thr172 AMPKα level normalized by β-actin. Phospho-AMPKα level was significantly increased after I/R in LETO but not in OLETF. (**C**) Representative blots for phospho-Ser555 and total ULK1. The blot of β-actin is identical to that in Fig. 3D because blots of both beclin-1 and ULK1 were from the same membrane. (**D**) Summary data for phospho-ULK1 level normalized by total ULK1. Phospho-ULK1 level was also elevated after I/R only in LETO. N = 8 in each group. (**E**) Representative blots for SIRT1 in renal tissues. (**F**) Summary data for SIRT1 protein level normalized by β-actin. In contrast to LETO, I/R failed to increase SIRT1 protein level in OLETF. N = 6 in each group. *P < 0.05. NS = not significant.

AMP-activated protein kinase (AMPK) activates autophagy by phosphorylating ULK1^38, 39^, and ischemia activates AMPK in cortical tubules^40^. To assess AMPK/ULK1 activity in the kidney tissue, immunoblotting was performed. Phospho-Thr172 AMPKα levels before ischemia were similar in LETO and OLETF. I/R significantly increased phospho-AMPKα levels in LETO but not in OLETF (Fig. 4A and B). The phosphorylation level of ULK1 at Ser555, one of the phosphorylation targets of AMPK for ULK1 activation^38^, was significantly increased by I/R in LETO but not in OLETF (Fig. 4C and D). SIRT1, an NAD^+^-dependent protein deacetylase, is reported to positively regulate AMPK activity via deacetylation of LKB1, an upstream AMPK activator^41^. In addition, SIRT1 is expressed in glomerular and proximal tubular cells in the kidney, and SIRT1 in the tubular cells is reported to be markedly downregulated in a model of T1DM^42^. Therefore, we hypothesized that downregulation of SIRT1 contributes to impaired AMPK activation in OLETF. Immunoblotting showed that SIRT1 protein level in the kidney was significantly upregulated after I/R in LETO, but not in OLETF (Fig. 4E and F). These findings suggest that impaired activation of the SIRT1/AMPK/ULK1 pathway contributed to impaired autophagic activation in OLETF.

**Figure 5:**
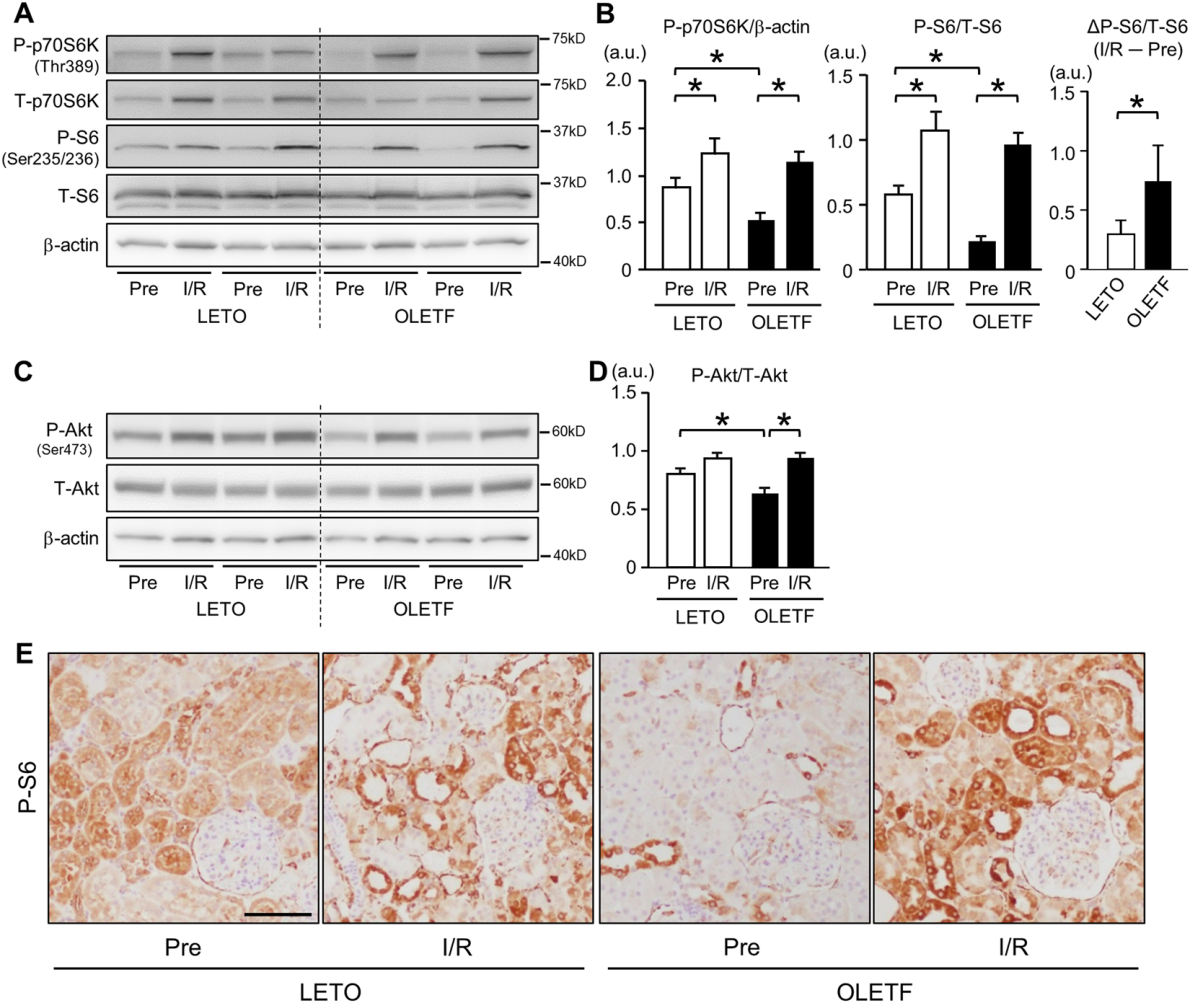
Analysis of Akt/mTORC1 signal pathways. Representative immunoblots (**A**) and summary data (**B**) for phospho-Thr389 and total p70S6 kinase (p70S6K), phospho-Ser235/236 and total S6, and actin in renal tissues before ischemia and 24 h after reperfusion in LETO and OLETF. Pre: pre-ischemia, I/R: 24 h after ischemia/reperfusion. (**B**) Before ischemia, phospho-p70S6K and phospho-S6 levels were reduced in OLETF. Levels of phospho-p70S6K and phospho-S6 were increased after I/R in both groups. The change in phospho-S6 level (ΔP-S6/T-S6) from the pre-ischemic period to 24 h after I/R was greater in OLETF than in LETO. (**C**) Representative immunoblots for phospho-Ser473 and total Akt. (**D**) Phospho-Akt level before ischemia was lower in OLETF than in LETO. I/R increased phospho-Akt levels in both OLETF and LETO, and phospho-Akt levels after I/R were similar in LETO and OLETF. N = 8 in each group. (**E**) Representative images of immunostaining for phospho-S6 in kidney sections before and 24 h after ischemia/reperfusion in LETO and OLETF. Scale bar, 100 μm. *P < 0.05.

We next examined whether mechanistic target of rapamycin complex 1 (mTORC1) signaling, a negative regulator of autophagy, is altered in OLETF. Basal levels of phospho-Thr389 p70S6 kinase (p70S6K) and phospho-Ser235/236 S6 were significantly lower in OLETF than in LETO, and I/R significantly increased the phosphorylation of p70S6K and S6 in both LETO and OLETF (Fig. 5A and B). Although levels of phospho-p70S6K and phospho-S6 after I/R were comparable in LETO and OLETF, the degree of change by I/R in phospho-S6 level was greater in OLETF (Fig. 5B, right panel). Total p70S6K protein level was also increased after I/R in both groups (Fig. 5A).

Phospho-Ser473 level of Akt, an upstream regulator of mTORC1, was significantly reduced in OLETF compared with that in LETO. However, I/R increased phospho-Akt levels in both groups, and there was no significant difference between phospho-Akt levels after I/R in LETO and OLETF (Fig. 5C and D).

Immunohistochemistry revealed that phospho-S6 was present mainly in tubular cells and parietal epithelial cells in OLETF before ischemia (Fig. 5E). As observed in immunoblotting, phospho-S6 staining was also reduced in OLETF before I/R, especially in proximal tubular cells. I/R increased phospho-S6-positive tubular cells in LETO and OLETF (Fig. 5E).

### Restoration of autophagy by rapamycin attenuated I/R-induced renal injury in OLETF

**Figure 6:**
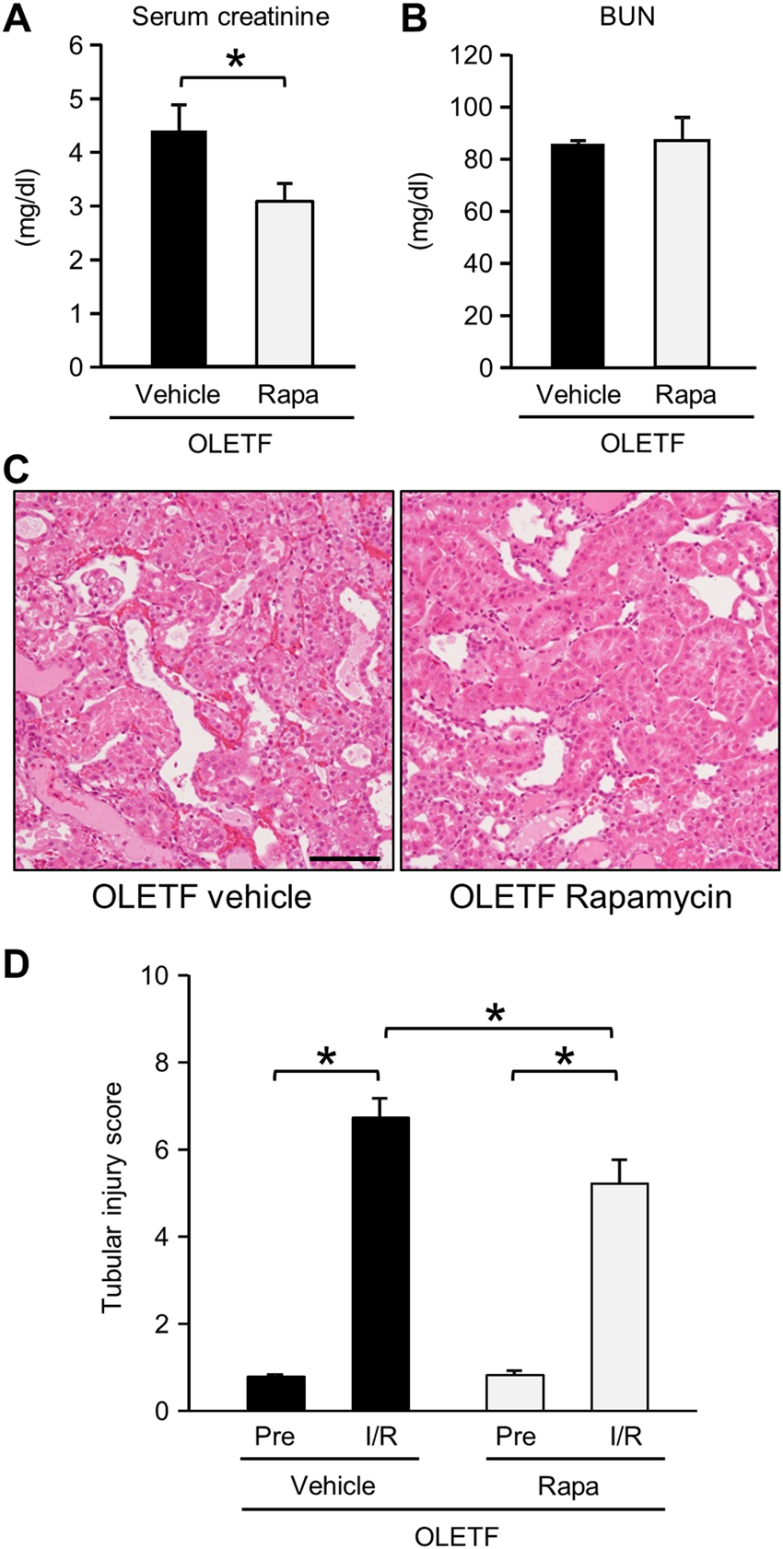
Effects of rapamycin on kidney injury after ischemia/reperfusion in OLETF. Levels of serum creatinine (**A**) and blood urea nitrogen (BUN) (**B**) 24 h after renal ischemia/reperfusion in OLETF treated with a vehicle (N = 7) or rapamycin (Rapa, 0.25 mg/kg, N = 7) 30 min prior to ischemia in OLETF. (**C**) Representative images of hematoxylin-eosin staining of renal tissues at 24 h after ischemia/reperfusion. (**D**) Tubular injury score before ischemia and 24 h after reperfusion in OLETF treated with vehicle or rapamycin. Pre: pre-ischemia, I/R: 24 h after ischemia/reperfusion. *P < 0.05. Scale bar, 100 μm.

**Figure 7:**
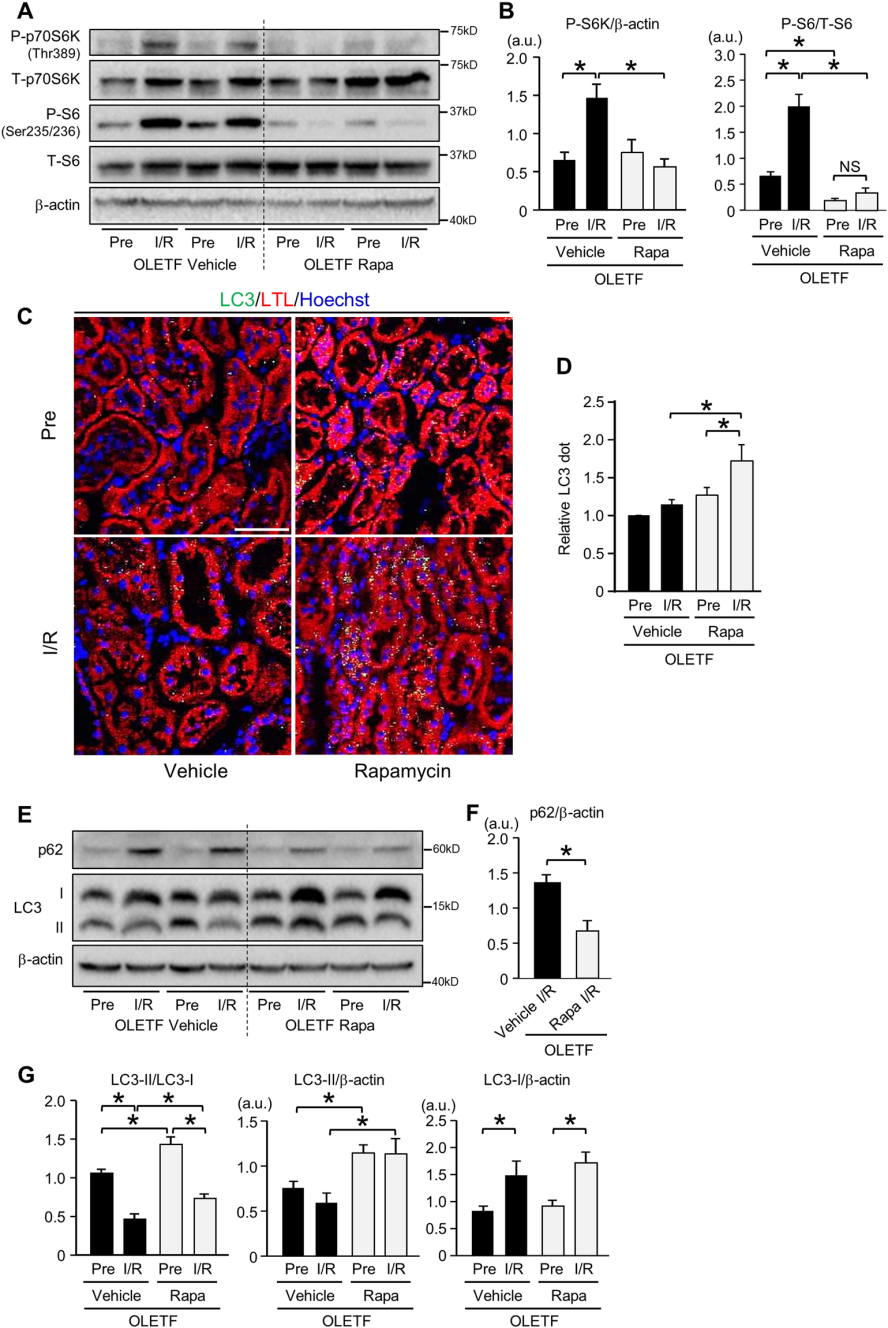
Restoration of autophagic response by rapamycin in OLETF. (**A**) Representative images of immunoblotting for phospho-Thr389 and total p70S6 kinase (p70S6K) and for phospho-Ser235/236 and total S6 of kidney tissues before ischemia and 24 h after reperfusion in OLETF pretreated with a vehicle (N = 7) or rapamycin (N = 7). Pre: pre-ischemia, I/R: 24 h after ischemia/reperfusion, Rapa; rapamycin-treated group. (**B**) I/R-induced increases in phospho-p70S6K and phospho-S6 levels were completely suppressed by rapamycin. (**C**) Representative immunofluorescence images of LC3 (green) and the proximal tubular marker lotus tetragonolobus lectin conjugated with Texas Red (LTL; red) in the kidneys from OLETF administered a vehicle or rapamycin. Nuclei were stained with Hoechst33342. (**D**) Summary data for LC3 dot level. (**E**) Representative blots for p62 and LC3. (**F**) Protein level of p62 after I/R was significantly reduced by rapamycin in OLETF. (**G**) LC3-II/LC3-I ratio after I/R was significantly increased by rapamycin, and the change was associated with a significant increase in LC3-II protein level. Scale bar, 50 μm. *P < 0.05.

Finally, we examined whether restoration of renal autophagy attenuates I/R-induced renal damage in OLETF. To activate autophagy, we chose an mTORC1 inhibitor rapamycin. OLETF were assigned to a vehicle group (n = 7) and a rapamycin group (n = 7). All of the rats were alive at 24 h after I/R. Rapamycin did not affect levels of blood glucose, glycoalbumin, total cholesterol, and triglyceride after I/R (Supplementary Table 2). Heart rate and blood pressure at 24 h after I/R were not changed by rapamycin treatment (Supplementary Table 3). Rapamycin modestly, but significantly decreased serum creatinine level after I/R to 3.08 ± 0.50 mg/dl compared with that in vehicle-treated animals (4.40 ± 0.21 mg/dl), though BUN level was unchanged (Fig. 6A and B). Tubular injury score after I/R was also significantly lower in rapamycin-treated OLETF than in vehicle-treated animals (Fig. 6C and D). Rapamycin completely suppressed elevation of phospho-p70S6K and phospho-S6 levels induced by I/R (Fig. 7A and B). Immunofluorescent labeling of LC3 revealed that rapamycin partially, but significantly, restored I/R-induced increase of autophagosomes in tubular cells in OLETF (Fig. 7C and D). Rapamycin significantly reduced p62 protein level after I/R (Fig. 7E and F) and increased LC3-II/LC3-I ratio and LC3-II level at baseline and after I/R (Fig. 7E and G).

## Discussion

In the present study, OLETF showed higher serum creatinine and BUN levels and higher tubular injury score after renal I/R than those in LETO. Such augmented AKI in OLETF was associated with significant suppression of the I/R-induced increase in autophagosomes in tubular cells and reduction in the LC3-II/LC3-I ratio. The augmentation of renal I/R injury in OLETF was mimicked in LETO treated with CQ, an inhibitor of autophagy, whereas CQ did not further increase I/R injury in OLETF. Conversely, rapamycin, a promoter of autophagy, significantly restored I/R-induced autophagic response and reduced both serum creatinine level and tubular injury score after renal I/R in OLETF. These findings support the hypothesis that impaired autophagic response in tubular cells underlies the aggravation of renal I/R injury by T2DM. This is the first study indicating the involvement of impaired autophagy in T2DM-induced aggravation of AKI.

We used OLETF at ages of 25–30 weeks when early stage of diabetic nephropathy had been developed (Table 1). Since multiple mechanisms are involved in diabetic nephropathy, there is the possibility that both autophagy-dependent and –independent mechanisms of renoprotection are impaired in OLETF with diabetic nephropathy. However, administration of CQ, an autophagy inhibitor, to LETO reproduced change in serum levels of creatinine and BUN and tubular injury score after I/R in OLETF (Fig. 1). On the other hand, CQ did not further augment I/R injury in OLETF, arguing against the possibility that the effect of CQ in LETO was due to toxicity unrelated to autophagy inhibition. Thus, autophagy-independent factors associated with diabetic nephropathy, if any, are unlikely to play a major role in increased renal susceptibility to I/R in OLETF.

As in earlier studies^13, 16–19^, I/R induced a significant increase in autophagosomes labelled by fluorescent LC3 in renal tubular cells in non-diabetic controls, LETO (Fig. 2). The increase in autophagosomes is explained by promotion of autophagosome formation, not by suppression of autophagosome clearance, because inhibition of autophagic flux by CQ further increased autophagosomes in the tubules after I/R (Fig. 2). In contrast to LETO, OLETF showed no significant increase in autophagosomes in renal tubular cells after I/R, indicating impaired activation of autophagy in response to I/R. This notion is consistent with lack of beclin-1 upregulation after I/R in OLETF (Fig. 3D and E).

The mechanism of I/R-induced activation of autophagy in the kidney has not been fully evaluated. Renal ischemia was reported to induce AMPK phosphorylation globally including tubular cells in rodents^40, 43^. Consistent with the earlier reports, we found that I/R significantly increased phosphorylation levels of both AMPKα and ULK1 in LETO (Fig. 4). Interestingly, mTORC1, which suppresses autophagy, was also activated after I/R (Fig. 5). Therefore, it is likely that AMPK-ULK1 is mainly responsible for I/R-induced activation of autophagy in renal tubular cells in LETO. However, such I/R-induced phosphorylation of AMPK and ULK1 was not observed in OLETF (Fig. 4). In addition, the increase in phospho-S6 level by I/R was significantly greater in OLETF than in LETO (Fig. 5). Since AMPK is known to suppress mTORC1 activity by phosphorylating tuberous sclerosis complex^44^, attenuated AMPK activation in OLETF may have led to hyperactivation of mTORC1. Taken together, these findings suggest that blunted autophagic response to I/R in OLETF is attributable to suppressed activity of AMPK/ULK1 and enhanced mTORC1 activation.

Attenuation of AMPK activation in the kidney of OLETF (Fig. 4) is consistent with findings in other animal models of diabetes mellitus^45, 46^, and the effect of diabetes mellitus on AMPK does not appear to be kidney-specific since we recently found that activation of myocardial AMPK in response to acute heart failure was impaired by T2DM^22^. How T2DM suppresses AMPK activity remains unclear. However, there are a few possible explanations. In the present study, SIRT1, a protein deacetylase, which activates AMPK activation^41^, was upregulated after I/R in LETO but not in OLETF (Fig. 4). The suppressed expression of SIRT1 is a possible mechanism of impaired AMPK activation in OLETF. In addition, attenuation of upstream AMPK kinase activity, enhanced activity of AMPK phosphatase, or an increase in inhibitory phosphorylation on AMPK at Ser485/491 are also possible mechanisms of the suppression of AMPK activity. However, since AMPK-Ser485 is a target site of Akt^47^, comparable phosphorylation levels of Akt-Ser473 in OLETF and LETO after I/R (Fig. 5) argue against the involvement of this regulation in T2DM-induced suppression of AMPK.

mTORC1 activity in the kidney was significantly increased after I/R (Fig. 5A and B), which is consistent with the previous reports^19, 32, 48, 49^. To induce renal I/R injury, we employed combination of right nephrectomy and 30-min ischemia/24 h-reperfusion in the left kidney. It was reported that unilateral nephrectomy itself led to mTORC1 activation in the contralateral kidney as a hypertrophic signaling even 24 h after surgery in mice^50^ and such an impact of unilateral nephrectomy in diabetic animals remains unclear. However, using the same one-kidney one-I/R model, Lieberthal *et al*.^32^ showed that phospho-p70S6K level was markedly higher in the kidney after I/R than that in the sham-operated remaining kidney after unilateral nephrectomy. In addition, significant activation of mTORC1 occurred in the post-ischemic kidney compared with sham in mouse models without nephrectomy^19, 49^. The findings suggest that I/R rather than nephrectomy largely accounts for mTORC1 activation observed in the kidney subjected to I/R in the present study.

The present study provided evidence indicating that impaired autophagy contributes to enhancement of renal I/R injury by diabetes mellitus and also showed that restoration of autophagy in the diabetic kidney is achievable by the use of rapamycin (0.25 mg/kg) (Fig. 7D). It is notable, however, that the renoprotective effect of rapamycin was relatively small while rapamycin almost completely suppressed mTORC1 activity. The relatively small impact of rapamycin on I/R injury might be due to incomplete restoration of autophagy. Not only modification of mTORC but also impaired AMPK activation are suggested to be responsible for suppressed autophagy in OLETF, and it is possible that AMPK activation needs to be restored for full activation of autophagy in OLETF. Selection of a dose of rapamycin for restoring autophagy in the kidney is actually difficult, and high doses of rapamycin can be detrimental to renal functions. In a study by Li *et al*.^19^, rapamycin treatment (5 mg/kg 1 day before and 2 days after I/R) reduced Ki67-positive injured proximal tubular cells after renal I/R in mice, and Grahammer *et al*.^51^ reported that inhibition of mTORC1 by deletion of raptor rather enhanced renal tubular injury and prevented proliferative response after I/R. In fact, in our pilot experiments, a high dose of rapamycin (2 mg/kg) administered prior to I/R did not afford significant renal protection in OLETF (data not shown). Strategy for restoring renoprotective autophagy warrants further investigation.

In the present study, rapamycin reduced tubular injury and serum creatinine level but not BUN level 24 h after I/R in OLETF. One possible explanation for the lack of significant change in BUN is that protection by rapamycin was not strong enough to decrease BUN level probably due to incomplete restoration of autophagic activation and suppression of the renal repair process afforded by mTORC1 as described above^51^. Another explanation is different time courses of BUN and creatinine level after I/R. Previous studies showed that serum BUN level reached the peak between 24 and 72 h following reperfusion while serum creatinine level peaked at 24 h after reperfusion^52, 53^. Therefore, it is possible that reduction of BUN level by rapamycin in OLETF could have been detected at 48~72 h after reperfusion.

p53 has been reported to be involved in I/R-induced AKI and cisplatin-induced AKI^54, 55^. Furthermore, a recent study by Peng *et al*.^8^ indicated that upregulation of p53 underlies the diabetes-induced enhancement of AKI. Using two models of T1DM, they showed that p53 protein level in the kidney was more upregulated after I/R in diabetic mice than in non-diabetic control mice. Interestingly, inhibition of p53 by pifithrin-α or knockdown of p53 by siRNA attenuated renal I/R injury assessed by serum creatinine level and histological tubular injury in diabetic mice^8^. However, we did not observe significant increase in p53 protein after I/R in the kidney of LETO or OLETF (Supplementary Fig. 2). Thus, the role of p53 in renal I/R injury might be different between T1DM and T2DM or between different protocols of I/R.

It remains unclear how autophagy exerts protection against I/R-induced renal injury. However, circumstantial evidence suggests that preservation of mitochondrial function is involved in the protection. Autophagy is important for re-cycling cellular components, including amino acids and lipids, and for elimination of damaged intracellular organelles, including mitochondria. Proximal tubular cells are rich in mitochondria and their function greatly depends on oxidative phosphorylation. A proximal tubule-specific defect of autophagy by Atg5 knockout has been shown to cause accumulation of deformed and damaged mitochondria^13, 18^. Furthermore, Kimura *et al*.^56^ showed that deletion of Atg5 significantly exaggerated metabolic stress on tricarboxylic acid cycle by cyclosporine A, leading to ATP depletion and enhanced production of reactive oxygen species (ROS), in proximal tubular cells. I/R not only reduces ATP-producing capacity of mitochondria but also increases ROS production^57^. Thus, preservation of energy substrates and mitochondrial function and elimination of cytotoxic ROS produced from damaged mitochondria are plausible mechanisms for renal protection afforded by autophagy. The contribution of such mechanisms needs to be examined by further investigations.

Although p62 protein level is often used as a marker of autophagic activity^35^, its level in whole kidney lysates was not useful for assessment of autophagic activity in the present study. The level of p62 was markedly increased after I/R in both LETO and OLETF without an inter-group difference (Fig. 3), though a difference in autophagic flux after I/R between the groups was indicated by analyses of LC3-positive autophagosomes (Fig. 2). Additionally, CQ did not change p62 protein level before and after I/R (data not shown). Consistent with the present results, earlier studies have shown that p62 protein increased in the kidney after I/R^49, 58^ and during cisplatin-induced acute injury^20^. Decleves *et al*.^58^ speculated that activity of autophagy during I/R was insufficient to clear p62 protein because p62 upregulation was suppressed by further promotion of autophagy by activating AMPK. In fact, promotion of autophagy by rapamycin reduced the level of p62 (Fig. 7E). However, changes in p62 level in OLETF cannot be explained by autophagy activity alone, because p62 protein level after I/R in OLETF, in which autophagic response was blunted, was similar to that in LETO (Fig. 3A and B). Another possible explanation for I/R-induced p62 upregulation is transcriptional activation of p62 by I/R-induced activation of Nrf2, a transcription factor targeting p62, in the kidney^59, 60^.

As a model of T2DM in the present study, we selected OLETF that is hyperphagic due to lack of functional cholecystokinin (CCK)-1 receptor. Zucker diabetic fatty (ZDF) rats, a model with defective leptin signaling, is also widely used, and suppressed activities of autophagy in the brain and myocardium have been shown in this model^61, 62^. However, since leptin reportedly modulates autophagy^63–65^, contribution of defective leptin signaling *per se* to modification of autophagy cannot be excluded in ZDF. Importantly, impaired autophagic activity has been reported for human samples; kidney biopsy samples^26^ and endothelial cells^66^ from diabetic patients showed reduced autophagic activity. Therefore, impaired autophagy is likely to be a common feature in T2DM regardless of genetic background or etiology.

In conclusion, T2DM impairs autophagic response to I/R in the kidney via suppression of SIRT1/AMPK/ULK1 signaling and enhancement of mTORC1, and the insufficient activation of autophagy during I/R contributes to aggravation of I/R-induced AKI by T2DM.

## Methods

### Animals and experimental protocol

The present study was conducted in strict accordance with the Guide for the Care and Use of Laboratory Animals published by National Research Council of the National Academies, USA (2011) and was approved by the Animal Use Committee of Sapporo Medical University. Male LETO and OLETF at ages of 25–30 weeks were used in all experiments. In a group of CQ-treated LETO and a group of CQ-treated OLETF, CQ (10 mg/kg/day, Sigma-Aldrich, St. Louis, MO, USA) was administered subcutaneously by osmotic minipumps (Alzet, Cupertino, CA, USA) for 1 week. The dose of CQ was selected on the basis of results in earlier reports^22, 29, 30^. Using separate groups of OLETF, we tested the effect of restoring autophagy by inhibition of mTORC1 on renal injury. Either rapamycin (0.25 mg/kg, LC laboratories, Woburn, MA) or its vehicle (0.25% Tween 80, 0.25% polyethylene glycol) was administered to OLTEF intraperitoneally 30 min before induction of renal I/R.

### Assessment of basal renal status in LETO and OLETF

To assess basal renal status, blood, urine, and kidney tissues were sampled from LETO and OLETF after fasting for 12 h. Rats were anesthetized with 2.5% isoflurane inhalation and supplemental oxygen, and blood was collected via a catheter cannulated into the carotid artery. After opening the abdominal cavity, the kidneys were removed, quickly weighed, washed in ice-cold saline, and then stored for histological analyses. Area of glomerular and mesangial matrix was determined in PAS staining by using Nikon NIS-elements software (Nikon, Japan). Mesangial area was selectively highlighted by using a threshold tool of NIS Elements, and this threshold was applied for all samples. Urine was aspirated from the urinary bladder.

### Induction of I/R in the kidney

Animals were fasted 12 h before the experiment. Rats were anesthetized with isoflurane as described above and placed on a heating pad to maintain rectal temperature at 37 °C. In a prone position, surgery was performed using a dorsal approach. The right kidney was gently pushed out of the body cavity and then removed. The removed right kidney was immediately placed in ice-cold saline and cut in halves. One of the halves was stored at −80 °C and the other was fixed in 10% formaldehyde. Next, the left kidney was exposed and its renal pedicle was occluded using a vascular clamp for 30 min to induce ischemia. The ischemic kidney was placed within the body cavity to avoid the effect of hypothermia on renal damage. After reperfusion by releasing the clamp, the muscle layers of the incision and skin wound were closed. After surgery, the animals were allowed to feed and drink water *ad libitum*. Sterilized instruments were used for the surgical preparation.

### Blood and kidney tissue sampling after renal I/R

At 24 h after reperfusion, rats were re-anesthetized with isoflurane. Blood pressure and heart rate were monitored, and blood samples were taken via a catheter placed in the carotid artery. The abdomen was opened, and the kidney was excised and immediately immersed in iced-cold saline. The kidney was halved for storing one half at −80 °C and for fixing the other half with 10% formaldehyde.

### Assessment of renal injury

Renal injury was assessed histologically with hematoxylin and eosin staining and with PAS staining. Pictures of kidney tubular cells in the corticomedullary area were randomly taken and were scored as previously reported^13^. In brief, for each of four types of damages in tubules (i.e., loss of brush border, tubular dilation, cast formation, and cell lysis), a score was given according to the percentage of damaged areas (i.e., 1, <25% damaged; 2, 25–50% damaged; 3, 50–75% damaged; and 4, more than 75% damaged), and scores of the four types of damages were summed as a score for each area of interest. The score in each kidney was calculated as the average of scores of fifteen areas under magnification of x200 in light microscopy. In each group, 7~10 kidneys were included for analysis.

### Immunofluorescence

Frozen sections of renal tissues were fixed with 4% paraformaldehyde, blocked with 5% bovine serum albumin in PBS, and permeabilized with 0.3% triton X-100. The sections were then incubated with anti-LC3A/B (Cell Signaling Technology, #12741, 1:50) overnight at 4 °C. The samples were then incubated with an Alexa Fluor 488 anti-rabbit IgG antibody (Invitrogen) for 1 h at room temperature. Nuclei and proximal tubular cells were stained with Hoechst33342 (Dojindo, Kumamoto, Japan) and LTL conjugated with Texas Red® (EY Laboratories, Inc., San Mateo, CA), respectively. We simultaneously conducted immunostaining for LC3 in one kidney section per rat from LETO-pre (i.e., right kidney removed before left kidney I/R), LETO-I/R (left kidney subjected to I/R), OLETF-pre, OLETF-I/R, LETO+CQ-pre and LETO+CQ-I/R groups. Sections from a kidney from each of the six treatment groups were taken at the same session of confocal imaging under the same settings. Five fields in each section were randomly selected, and LC3 positive areas per field were averaged. Then, ratio of average LC3 dot level (LC3 positive area) to average LC dot level in LETO-Pre was calculated for each kidney as “relative LC3 dot number”. Finally, the “relative LC3 dot numbers” in 4~5 independent kidneys from each group were averaged. Fluorescence images were obtained using ConfoCor3LSM510META (Zeiss) under the same conditions. The percentage of area of LC3 dots was analyzed in five randomly selected fields in the each kidney, and 4~5 kidneys in each group were included for comparison among groups.

### Immunoblotting

Frozen tissue samples were homogenized in ice-cold buffer (CelLytic™ MT Cell Lysis Reagent, Sigma-Aldrich) including protease and phosphatase inhibitor cocktails (Nacalai Tesque, Inc., Kyoto, Japan). The homogenate was centrifuged at 15,000 *g* for 15 min at 4 °C to obtain the supernatant. Equal amounts of protein were analyzed by immunoblot assays using specific antibodies (Supplementary Table 4). Intensities of individual bands were quantified by using Image J software (National Institutes of Health).

### Statistical analyses

Data are presented as means ± SEM. Statistical significance was determined using unpaired Student’s two-tailed t-test for two data sets. Differences between four treatment groups were assessed by one-way analysis of variance (ANOVA) followed by the Student-Newman-Keuls *post hoc* test for multiple comparisons. Two-way repeated measures ANOVA and Student-Newman-Keuls *post hoc* test were used to analyze differences in data between pre-I/R and after I/R in LETO and OLETF. For all tests, p < 0.05 was considered statistically significant. All analyses were performed with SigmaStat (Systat, San Jose, CA, USA).

## Electronic supplementary material

Supplementary Data
