# Supplementary material for: Suppressed autophagic response underlies augmentation of renal ischemia/reperfusion injury by type 2 diabetes
Source: Sci Rep. 2017 Jul 13;7:5311. doi: 10.1038/s41598-017-05667-5 (PMC5509657; doi:10.1038/s41598-017-05667-5)
Supplement: Supplementary file 1 — Supplementary Data [file 41598_2017_5667_MOESM1_ESM.pdf]

Supplementary Information

Suppressed autophagic response underlies augmentation of renal ischemia/reperfusion injury by type 2 diabetes.

Shingo Muratsubaki, Atsushi Kuno†, Masaya Tanno, Takayuki Miki, Toshiyuki Yano, Hirohito Sugawara, Satoru Shibata, Koki Abe, Satoko Ishikawa, Kouhei Ohno, Yukishige Kimura, Yuki Tatekoshi, Kei Nakata, Wataru Ohwada, Masashi Mizuno, Tetsuji Miura

Department of Cardiovascular, Renal and Metabolic Medicine, †Department of Pharmacology,
Sapporo Medical University School of Medicine, Sapporo, Japan.

Supplementary Table 1. Hemodynamic parameters 24 h after renal ischemia/reperfusion

	LETO (N=12)	OLETF (N=12)	LETO+CQ (N=8)	OLETF+CQ (N=10)
Heart rate (bpm)	349±4	277±12*	307±10*	260±9[#]
Systolic blood pressure (mmHg)	104±3	104±2	108±3	117±3[#†]
Diastolic blood pressure (mmHg)	72±4	70±3	66±2	79±2[#†]

Values are means ± SEM. *p<0.05 vs. LETO, [#]P<0.05 vs. LETO+CQ, [†]P<0.05 vs. OLETF

Supplementary Table 2. Effect of rapamycin on metabolic parameters after renal ischemia/reperfusion.

	OLETF+ Vehicle (N=7)	OLEFT+ Rapamycin (N=7)
Body weight (g)	641±12	650±16
Blood glucose (mg/dl)	231±21	234±20
Glycoalbumin (%)	17.2±3.0	14.0±1.3
Total cholesterol (mg/dl)	144±9	155±15
Triglyceride (mg/dl)	147.6±48.5	148.0±33.0

Values are means ± SEM.

Supplementary Table 3. Effects of rapamycin on hemodynamic parameters after renal ischemia/reperfusion

	OLETF+ Vehicle (N=7)	OLEFT+ Rapamycin (N=7)
Heart rate (bpm)	306±14	285±10
Systolic blood pressure (mmHg)	122±4	121±3
Diastolic blood pressure (mmHg)	85±7	78±3

Values are means ± SEM.

Supplementary Table 4. Antibodies used in the present study

Antibodies	Source	Cat. No.	dilution
Guinea pig anti-polyclonal p62	Progen	GP62-C	1:1000
Rabbit monoclonal anti-LC3A/B	Cell Signaling	#12741	1:1000
Rabbit polyclonal anti-phospho-Thr389-p70S6K	Cell Signaling	#9205	1:1000
Rabbit polyclonal anti-p70S6K	Cell Signaling	#9202	1:1000
Rabbit polyclonal anti-phospho-Ser235/236-S6	Cell Signaling	#2211	1:1000
Rabbit polyclonal anti-S6	Cell Signaling	#2217	1:1000
Rabbit polyclonal anti-phospho-Ser473-Akt	Cell Signaling	#9271	1:1000
Rabbit polyclonal anti-Akt	Cell Signaling	#9272	1:1000
Rabbit monoclonal anti-phospho-Thr172 AMPKα	Cell Signaling	#2535	1:1000
Rabbit polyclonal anti-AMPKα	Cell Signaling	#2532	1:1000
Rabbit monoclonal anti-phospho-Ser555-ULK1	Cell Signaling	#5869	1:1000
Rabbit monoclonal anti-ULK1	Abcam	ab128859	1:1000
Rabbit monoclonal anti-Beclin-1	Cell Signaling	#3495	1:1000
Mouse monoclonal anti-p53 (1C12)	Cell Signaling	#2524	1:1000
Mouse monoclonal anti-SIRT1 (19A7AB4)	Abcam	ab110304	1:2000
Mouse monoclonal anti-β-actin	Sigma-Aldrich	A5316	1:10000

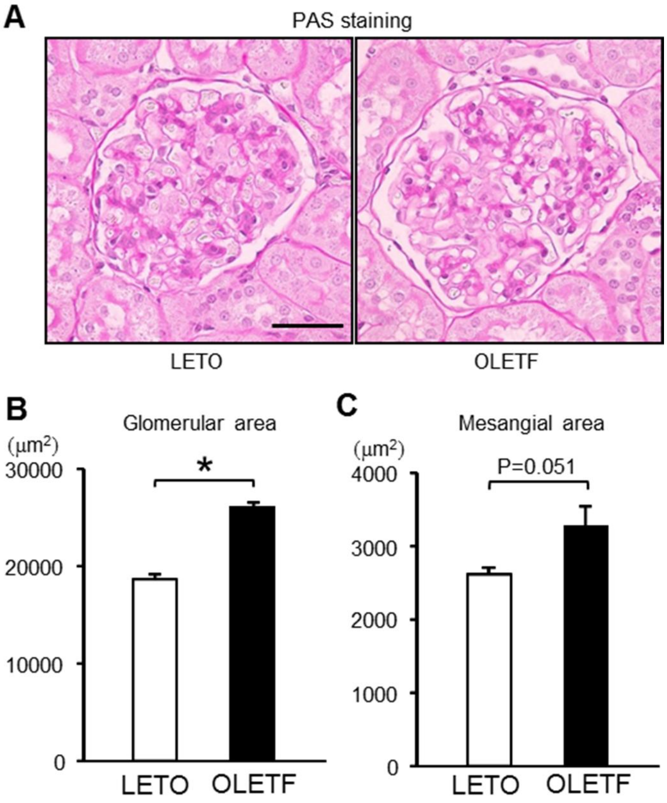

Supplementary Figure 1. Sizes of glomerular areas and mesangial areas in LETO and OLETF. Representative glomeruli stained with PAS (A) and group mean data for glomerular area size (B) and for mesangial area size (C). Fifteen glomeruli were randomly selected in each kidney sample and sizes of glomerular and mesangial areas were determined by Nikon NIS-elements software. N = 5 in each group. *P<0.05. Scale bar, 50 μm.

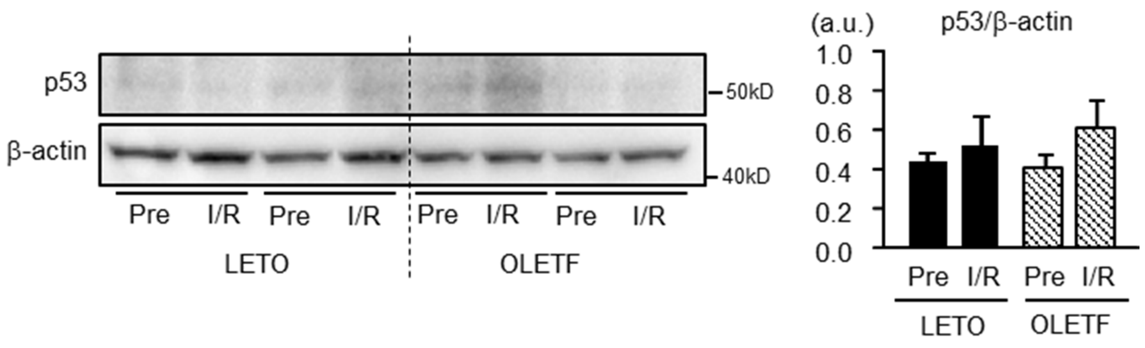

Supplementary Figure 2. Protein levels of p53 in LETO and OLETF before and after renal ischemia/reperfusion.Left panel: Representative immunoblots for p53 before ischemia (Pre) and 24 h after ischemia/reperfusion (I/R). Right panel: group mean data for p53 protein level normalized by β-actin level (n = 8 in each treatment group).
